# Transcriptome and metabolome reveal the accumulation of secondary metabolites in different varieties of *Cinnamomum longepaniculatum*

**DOI:** 10.1186/s12870-022-03637-2

**Published:** 2022-05-18

**Authors:** Xin Zhao, Yue Yan, Wan-hai Zhou, Rui-zhang Feng, Yong-kang Shuai, Li Yang, Meng-jie Liu, Xiu-yan He, Qin Wei

**Affiliations:** 1 Faculty of Agriculture, Forestry and Food Engineering, YiBin University, Yibin, 644000 Sichuan People’s Republic of China; 2 Sichuan Oil Cinnamon Engineering Technology Research Center, Yibin, 644000 Sichuan People’s Republic of China

**Keywords:** *Cinnamomum longepaniculatum*, Secondary metabolism, Flavonoids, Terpenoids

## Abstract

**Background:**

*Cinnamomum longepaniculatum* (Gamble) N. Chao ex H. W. Li, whose leaves produce essential oils, is a traditional Chinese medicine and economically important tree species. In our study, two *C*. *longepaniculatum* varieties that have significantly different essential oil contents and leaf phenotypes were selected as the materials to investigate secondary metabolism.

**Result:**

The essential oil content and leaf phenotypes were different between the two varieties. When the results of both transcriptome and metabolomic analyses were combined, it was found that the differences were related to phenylalanine metabolic pathways, particularly the metabolism of flavonoids and terpenoids. The transcriptome results based on KEGG pathway enrichment analysis showed that pathways involving phenylpropanoids, tryptophan biosynthesis and terpenoids significantly differed between the two varieties; 11 DEGs (2 upregulated and 9 downregulated) were associated with the biosynthesis of other secondary metabolites, and 12 DEGs (2 upregulated and 10 downregulated) were related to the metabolism of terpenoids and polyketides. Through further analysis of the leaves, we detected 196 metabolites in *C. longepaniculatum.* The abundance of 49 (26 downregulated and 23 upregulated) metabolites differed between the two varieties, which is likely related to the differences in the accumulation of these metabolites. We identified 12 flavonoids, 8 terpenoids and 8 alkaloids and identified 4 kinds of PMFs from the leaves of *C. longepaniculatum*.

**Conclusions:**

The combined results of transcriptome and metabolomic analyses revealed a strong correlation between metabolite contents and gene expression. We speculate that light leads to differences in the secondary metabolism and phenotypes of leaves of different varieties of *C. longepaniculatum*. This research provides data for secondary metabolite studies and lays a solid foundation for breeding ideal *C. longepaniculatum* plants.

**Supplementary Information:**

The online version contains supplementary material available at 10.1186/s12870-022-03637-2.

## Background

Plants can synthesize a large diversity of secondary metabolites, which are notable for their beneficial biological activities that help plants colonize diverse and challenging environments. Secondary metabolites include major groups such as flavonoids, terpenoids, and alkaloids. Flavonoids are low-molecular-weight secondary metabolites that have antioxidant, antiproliferative [1, 2], inflammatory response-regulating [3] and antilithiatic [4] effects. Many studies have shown that flavonoids are synthesized in plants via the phenylpropanoid and acetate–malonate metabolic pathways [5]. Many flavonoid biosynthesis-related genes have been studied in depth, including those encoding phenylalanine ammonia lyase (PAL), cinnamate 4-hydroxylase (C4H), 4-coumaroyl CoA ligase (4CL) and MYB transcription factors [6]. In addition, flavonoids can negatively regulate auxin transport [7]. Polymethoxyflavones (PMFs) constitute a group of flavonoids that exert beneficial biological activities for humans, such as anticancer [8], antilipogenic, antitumor [9], regulate gut microbiome [10], and anti-inflammatory activities [11]. Since essential oils have similar functions, we hypothesize that there are PMFs in *Cinnamomum longepaniculatum*, but there are few reports about which flavonoids are present in the leaves of *C. longepaniculatum*.

Terpenoids (isoprenoids) are a class of important chemicals produced by plants [12, 13]. Many terpenoids have a long history of being used as flavour-enhancing compounds, pharmaceuticals, insecticides, and industrial compounds [14]. Terpenoids in plants can be divided into monoterpenes (C10), sesquiterpenes (C15), diterpenes (C20), triterpenes (C30), tetraterpenes (C40) and polyterpenes [15]. Plants use two independent pathways to produce terpenoids: the cytosolic mevalonic acid (MVA) pathway, and the plastidial methylerythritol phosphate (MEP) pathway [16–18]. During the last few decades, there has been increasing interest in the terpenes of *Cinnamomum* plants, mainly focusing on analysis of the main compounds of essential oil extracted from leaves [12, 14]. Many studies have attempted to evaluate the antibacterial activity of terpenoids that constitute essential oils [19, 20]. In recent years, many studies have focused on analysing the transcriptomes related to specific terpenoids in leaf metabolic pathways and their regulatory mechanisms [21] and the differential accumulation of terpenoids in different *Cinnamomum* chemotypes [22]. However, combined metabolome and transcriptome profiling of terpenoids in different varieties of *C. longepaniculatum* has not been reported.

*Cinnamomum longepaniculatum* (Gamble) N. Chao ex H. W. Li [23] is an evergreen tree species [24]. *C. longepaniculatum* is extensively cultivated in southwestern China, especially in the Yibin region (Sichuan, China). *C. longepaniculatum* leaves and twigs are harvested for essential oil extraction. Research on essential oils has mainly focused on their composition [25], especially their antibacterial activities [26] and endophytic fungi [27]. Terpenoids (monoterpenes and sesquiterpenes) produced during plant secondary metabolism are the major components of essential oils [19]. However, we know little about other secondary metabolites present in the leaves of *C. longepaniculatum*.

In this study, two different varieties of *C. longepaniculatum*, namely, CLH and CLL, were selected as the materials to study the secondary metabolite differences. Previous research has shown that the production of essential oils significantly differs among varieties, so we used transcriptomic and metabolomic data from functional leaves from different varieties to investigate their secondary metabolism. This research not only provides data for *C. longepaniculatum* secondary metabolite studies but also lays a solid foundation for breeding ideal *C. longepaniculatum* plant types.

## Results

### Leaf essential oil content and phenotypic changes of different varieties

**Fig. 1:**
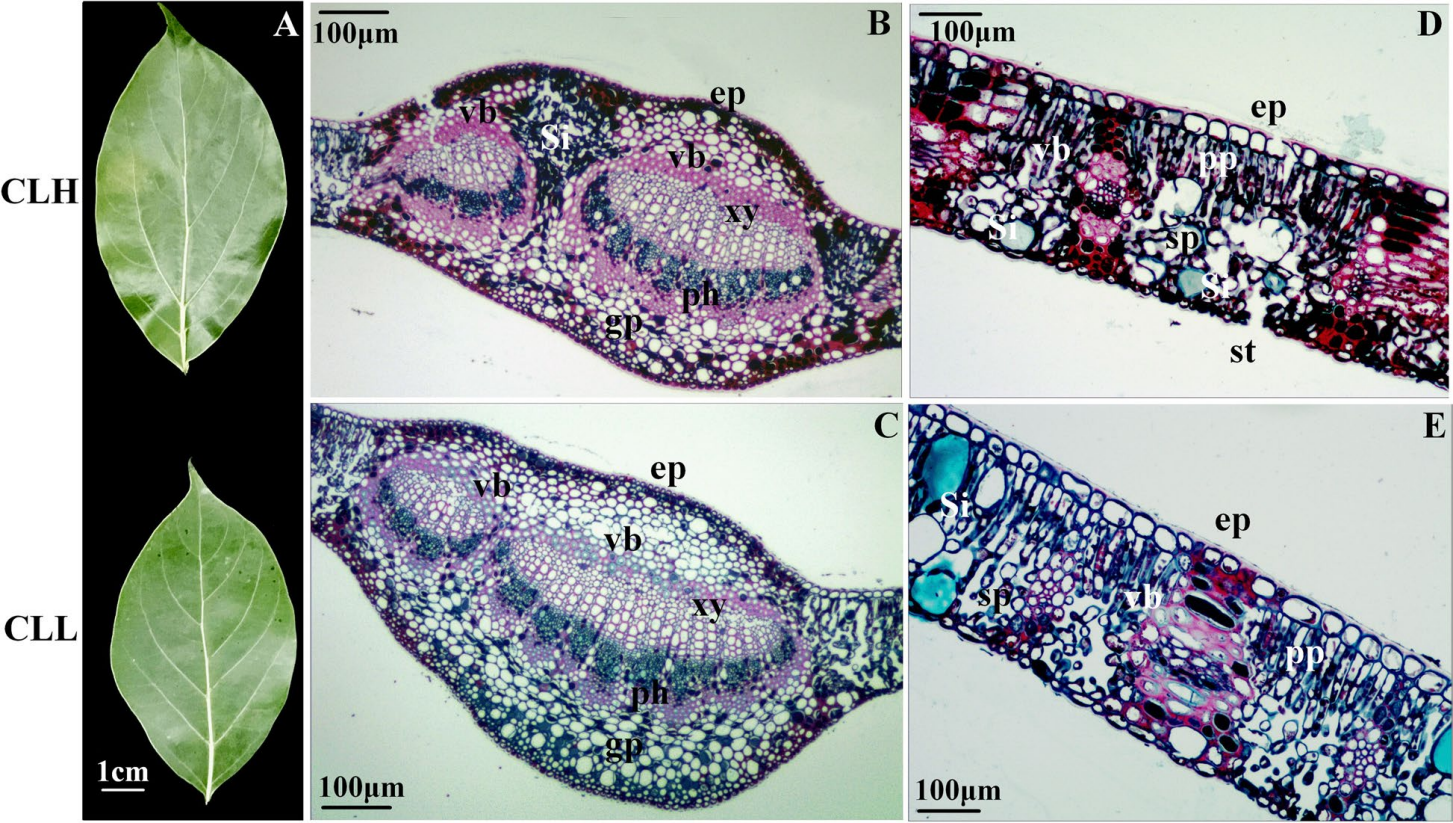
Leaf of *C*. *longepaniculatum*. **A** The phenotypical characteristics of different varieties. The midrib cross-section of CLH (**B**) and CLL (**C**). Blade organization is showing dorsiventral mesophyll of different varieties (**D, E**). ep, epidermis; pp., palisade parenchyma; si, idioblastsc; sp., spongy parenchyma; vb, vascular bundle; gp, ground parenchyma; ph, phloem; vb, vascular bundles; xy, xylem; st, stoma. Scale bar = A (1 cm), B, C, D and E (100 μm)

We sampled fresh leaves from different varieties for three years and extracted essential oils in the same way. Under the same climatic conditions, the essential oil contents of the different varieties of *C. longepaniculatum* were different. The results showed that the essential oil content of CLH was significantly higher than that of CLL (Table 1). Many differences in the leaves of the two varieties were detected. The leaf area of CLH was greater than that of CLL, but the difference was not significant (Fig. 1A). We compared cross-sections of leaves from the different varieties by paraffin sectioning. The cross-section of the midrib revealed a biconvex shape, and the average cross-section area for CLH was 82.94 μm^2^, while that for CLL was 72.82 μm^2^. We also measured the leaf thickness, and the average for CLH (226.57 μm) was significantly greater than that for CLL (189.10 μm). Many ground parenchyma (gp) cells were observed on the veins of the adaxial sides of the leaves. The vascular system was composed of two vascular bundles, which were arranged in the open arc shape, and the middle was very large. Secretory idioblasts (Si) were present between the midrib and lateral vein of the CLH leaves, but these were not present in the CLL leaves (Fig. 1B, C). The mesophyll comprised one layer of the epidermis. Small collateral vascular bundles were bordered by the endoderm and penetrate through the spongy parenchyma (Fig. 1D, E).

**Table 1:** Essential Oil content in leaves of two varieties in different years (%)

Different varieties	Years		
	2019	2020	2021
CLH	1.7199 ± 0.02a	1.9206 ± 0.03a	1.9113 ± 0.01a
CLL	1.2970 ± 0.03b	1.4416 ± 0.02b	1.3123 ± 0.02b

### Analysis of transcriptomic data

To explore the molecular mechanisms underlying secondary metabolism, we compared transcriptome between the different varieties. A robust data set was generated after data processing: 48.63 million and 46.68 million high-quality reads were obtained from CLH and CLL, respectively; moreover, 7.34 and 7.05 Gb were obtained, and the GC contents were 45.59 and 45.74%, respectively. The sequencing statistics of the 6 RNA generated libraries are listed in Table 2. BUSCO analysis showed that the completeness of *C. longepaniculatum* leaf transcriptome was 95.2%, indicating the excellent continuity of the assembly (Table 3). To compare the gene expression in CLH with that in CLL, Venn diagram analysis showed that 34,503 genes were present in both comparison groups (Fig. S1).

**Table 2:** The sequencing statistics for 6 RNA libraries of *C. longepaniculatum*

Sample	CLH_1	CLH_2	CLH_3	CLL_1	CLL_2	CLL_3
Raw reads	44,061,552	50,122,094	51,718,942	49,379,184	45,048,422	45,636,610
Raw bases	6,653,294,352	7,568,436,194	7,809,560,242	7,456,256,784	6,802,311,722	6,891,128,110
Clean reads	43,776,584	49,761,596	51,338,780	48,950,688	44,744,566	45,296,960
Clean bases	6,433,300,702	7,335,310,435	7,561,568,161	7,229,138,023	6,594,257,446	6,650,661,594
Q30 (%)	93.44	92.97	93.21	93.18	93.65	93.36
GC content (%)	45.64	45.54	45.58	46.13	45.47	45.63
Mapped reads	34,075,222	39,118,058	40,296,402	38,020,826	34,865,230	34,879,358
Mapped ratio(%)	77.84	78.61	78.49	77.67	77.92	77.00

**Table 3:** Transcriptome-optimized assembly results evaluation of *C. longepaniculatum*

	Unigene	Transcript
Total number	169,860	244,103
Total base	131,791,731	233,224,657
Largest length (bp)	17,036	17,036
Smallest length (bp)	201	201
Average length (bp)	775.88	955.44
N50 length (bp)	1259	1680
Fragment mapped percent (%)	61.447	77.973
GC percent (%)	43.03	42.5
TransRate score	0.27732	0.35112
BUSCO score (%)	73.0	89.4

**Fig. 2:**
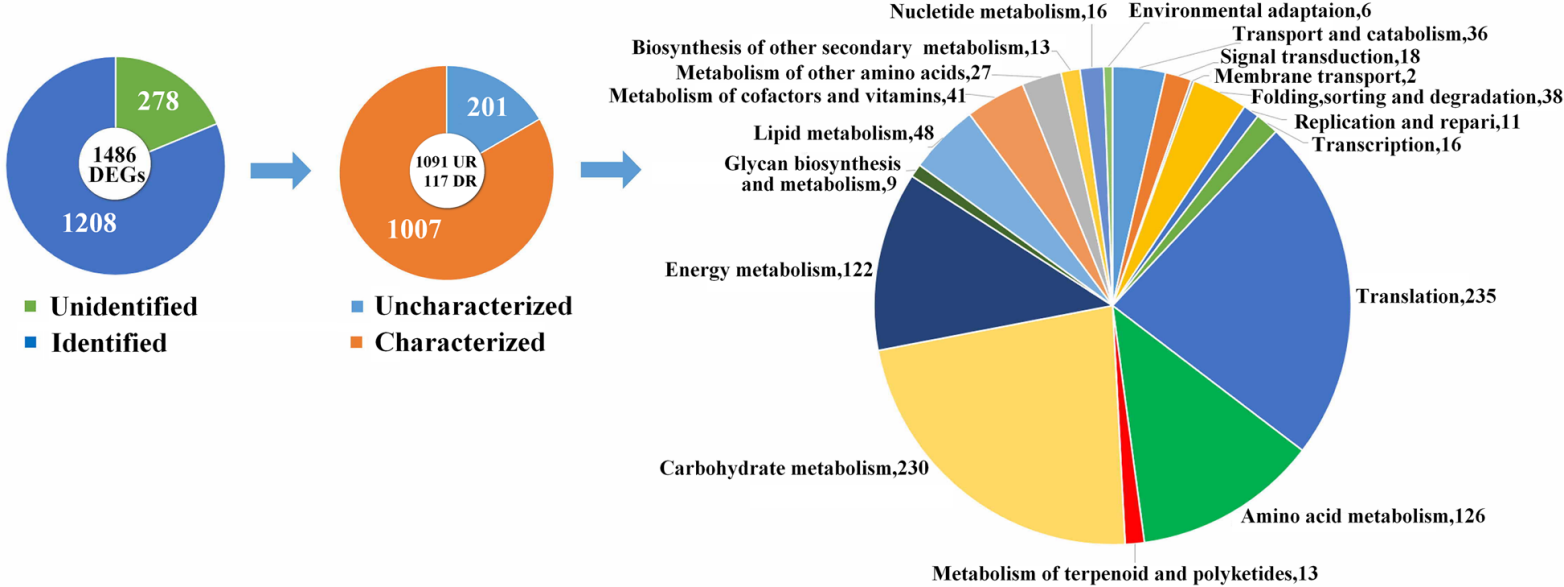
Distribution and classification of differentially expressed genes (DEGs) in CLH compared with CLL

After comparing CLH and CLL, we identified 1486 differentially expressed genes (DEGs) (*P* value < 0.05, fold-change (FC)>2), all of which may be related to the differences in secondary metabolism. Among these DEGs, 1357 were upregulated, and 129 were downregulated; the corresponding FCs and P value are listed in Table S1. Through matching with the Gene Ontology (GO), SwissProt, Clusters of Orthologous Groups of proteins (COG) and Pfam database information, 1208 DEGs (%) were identified (Fig. 2). According to functional classification, 1007 DEGs with known functions were further partitioned into 5 initial categories, and 645 DEGs were found to be involved in metabolism (Table S2). Then, we performed Kyoto Encyclopedia of Genes and Genomes (KEGG) enrichment analysis of the second KEGG pathway category, which revealed the following pathways: transport and catabolism (36); signal transduction (18); membrane transport (2); folding, sorting and degradation (38); replication and repair (16); transcription (11); translation (235); amino acid metabolism (126); biosynthesis of other secondary metabolites (13); carbohydrate metabolism (230); energy metabolism (122); glycan biosynthesis and metabolism (9); lipid metabolism (48); metabolism of cofactors and vitamins (41); metabolism of other amino acids (27); metabolism of terpenoids and polyketides (13); nucleotide metabolism (16); and environmental adaptation (6) (Fig. 2). The KEGG analysis showed that the phenylpropanoid, tryptophan biosynthesis and terpenoid pathways were significantly different between the two varieties (Table S3). We were more interested in the pathways related to secondary metabolism: biosynthesis of other secondary metabolites (2 upregulated and 9 downregulated DEGs) and metabolism of terpenoids and polyketides (2 upregulated and 10 downregulated DEGs). In addition, there were 3 KEGG pathways that were related to light: photosynthesis (map00195, 35 DEGs), photosynthesis-antenna proteins (map00196, 18 DEGs), and oxidative phosphorylation (map00190, 29 DEGs) (Table S2).

### Metabolome profiling of different varieties

**Fig. 3:**
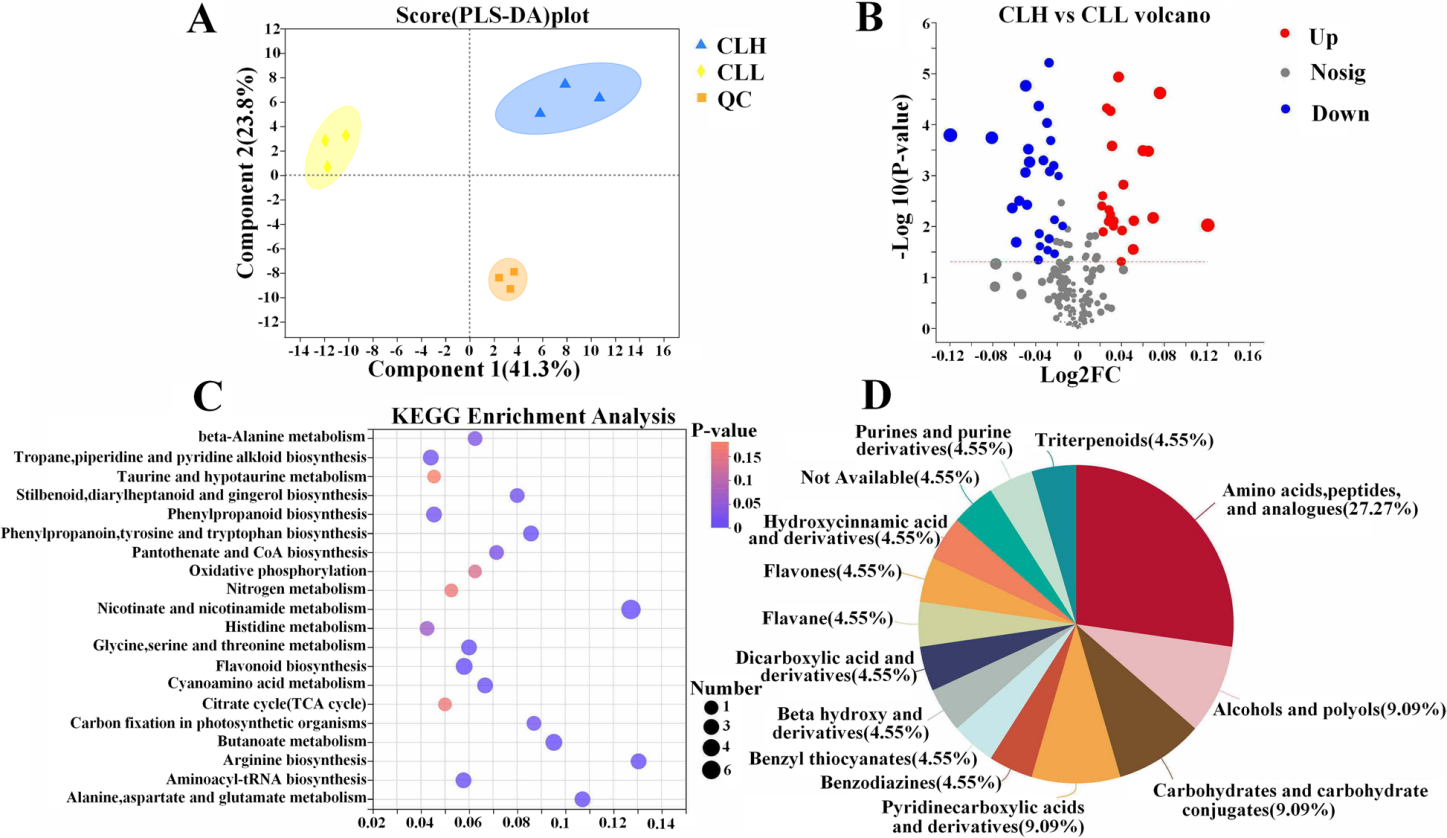
Metabolome data analysis. **A** The PLS-DA analysis. **B** Comparison of gene expression levels between the CLH and CLL according to the OPLS-DA model. **C** Scatterplot of the KEGG pathway enriched by different metabolites for the CLH versus CLL group. The vertical axis represents the name of the pathway, and the horizontal axis represents the *P* value. The size and color of bubbles indicate the number and degree of enrichment of different metabolites, respectively. **D** The differentially accumulated metabolites are mapped to the Human Metabolome Database (HMDB)

Metabolites were extracted from leaf samples (three replicates) for each experimental group and were analysed by GC–MS. Partial least squares discrimination analysis (PLS-DA) resulted in the division of the total variation into two major components (Component 1 and Component 2), which contributed 41.3 and 23.8% of the variation, respectively (Fig. 3A). In total, we detected 196 metabolites from *C. longepaniculatum* (Table S4): 147 were matched with KEGG database information, and 92 were characterized in the library. The analysis of the function of the metabolites revealed nine main aspects: amino acid metabolism (34), biosynthesis of other secondary metabolites (24), carbohydrate metabolism (24), energy metabolism (8), lipid metabolism (9), metabolism of cofactors and vitamins (17), metabolism of other amino acids (11), metabolism of terpenoids and polyketides (6), and nucleotide metabolism (8) (Fig. S2).

To further determine the differences in metabolites between CLH and CLL, under the thresholds of a variable importance in projection (VIP)_pred_orthogonal projections to latent structures-discriminant analysis (OPLS-DA) > 1.0, a FC ≥ 1 or ≤ 1, and a *P* value < 0.05, 49 (26 decreased and 23 increased) proteins were found to be differentially accumulated. The screening results were visualized by volcano plots (Fig. 3B). The forty-nine differentially accumulated metabolites (DAMs) were mapped to KEGG pathways (Fig. 3C). The DAMs were mainly enriched in flavonoid biosynthesis (4); phenylpropanoid biosynthesis (3); phenylalanine, tyrosine and tryptophan biosynthesis (3); phenylalanine metabolism (3); and stilbenoid, diarylheptanoid and gingerol biosynthesis (2). Some DAMs related to oxidative phosphorylation and carbon fixation in the photosynthetic organism pathway were also found (Fig. 3C). We identified 12 flavonoids, 8 terpenoids and 8 alkaloids in the leaves of *C*. *longepaniculatum* (Table 4). Through searching against the Human Metabolome Database (HMDB) (https://hmdb.ca/), we found that 22 DAMs had matches (Fig. 3D).

**Table 4:** The contents of flavonoids, terpenoids and alkaloids in two varieties leaves of *C*. *longepaniculatum* (ng/mg)

Secondary metabolites			
Flavonoids		**CLH**	**CLL**
Flavones	Apigenin	115.34 ± 1.24a	102.60 ± 2.12b
	chrysin	5.98 ± 0.78a	6.81 ± 0.93a
	Acacetin	594.81 ± 3.56a	571.06 ± 2.15b
Flavanones	Neohesperidin	256.28 ± 6.33b	433.87 ± 5.29a
Flavan 3-ols	epicatechin	5.60 ± 2.58b	15.77 ± 1.56a
	catechin	372.61 ± 6.46b	478.33 ± 7.52a
Dihyroflavonols	taxifolin	71.40 ± 1.69a	73.92 ± 2.71a
Flavonols	kaempferol	137.53 ± 3.68b	155.36 ± 4.32a
PMFs	3,4′,5,6,7-Pentamethoxyflavone	0.96 ± 0.36a	1.51 ± 0.25a
	5-Hydroxy-2′,4′,7,8-Tetramethoxyflavone	7.53 ± 0.19b	8.85 ± 0.32a
	3-Hydroxy-3′,4′-Dimethoxyflavone	0.47 ± 0.21b	0.71 ± 0.16a
	3,7-Dihydroxyflavone	132.20 ± 2.36b	176.99 ± 5.23a
Terpenoids			
Cyclic monoterpenes	terpineol	749.98 ± 5.36b	983.32 ± 4.33a
	limonene	1.38 ± 0.56a	1.57 ± 0.28a
Linear monoterpenes	beta-myrcene	14.34 ± 0.43b	18.86 ± 0.59a
Linear diterpenes	phytol	190.58 ± 5.29a	200.23 ± 4.76a
Sesquiterpenoids	farnesal	116.88 ± 1.83a	118.21 ± 1.95a
Liner triterpenes	squalene	4.23 ± 0.45a	2.97 ± 0.13b
Carotenoids and apocarotenoids	beta-ionone	47.64 ± 2.71a	32.50 ± 1.68b
Steroids	beta-sitosterol	168.32 ± 1.86b	179.44 ± 2.55a
Alkaloids			
Piperidine alkaloids	pipecolic acid	17.59 ± 2.32b	24.76 ± 1.37a
Quinoline alkaloids	2,3-butanediol	93.75 ± 1.99b	114.67 ± 2.84a
Pyridine alkaloids	D-allose	2143.92 ± 3.26a	1908.26 ± 4.68b
	nicotinic acid	4.72 ± 0.29a	3.15 ± 0.27b
	hypoxanthine	212.25 ± 5.49b	247.30 ± 6.89a
	6-hydroxynicotinic acid	7.78 ± 1.13b	11.80 ± 0.94a
Indole alkaloids	melatonin	15.06 ± 0.29a	14.57 ± 0.73a
Isoquinoline alkaloids	Thebaine	24.20 ± 1.28b	27.24 ± 0.97a

### Changes in bioactive substances in the leaves of *C. longepaniculatum*

Through metabolomic analysis (Table 4), we identified 5 types of flavonoids in *C. longepaniculatum* leaves: flavones, flavanones, flavan-3-ols, dihydroflavonols, flavonols, and PMFs. Among them, PMFs were identified for the first time in *C. longepaniculatum* leaves, of which there were 4 kinds of compounds present: 3,4′,5,6,7-pentamethoxyflavone, 5-hydroxy-2′,4′,7,8-tetramethoxyflavone, 3-hydroxy-3′,4′-dimethoxyflavone, and 3,7-dihydroxyflavone. We identified 7 types of terpenoids: terpineol, limonene, beta-myrcene, phytol, farnesal, squalene, beta-ionone, and beta-sitosterol. Alkaloids including pipecolic acid, 2,3-butanediol, D-allose, nicotinic acid, hypoxanthine, 6-hydroxynicotinic acid, melatonin, and thebaine were also identified for the first time in *C. longepaniculatum* leaves.

### Correlation analysis between the transcriptomic and Metabolomic data

**Fig. 4:**
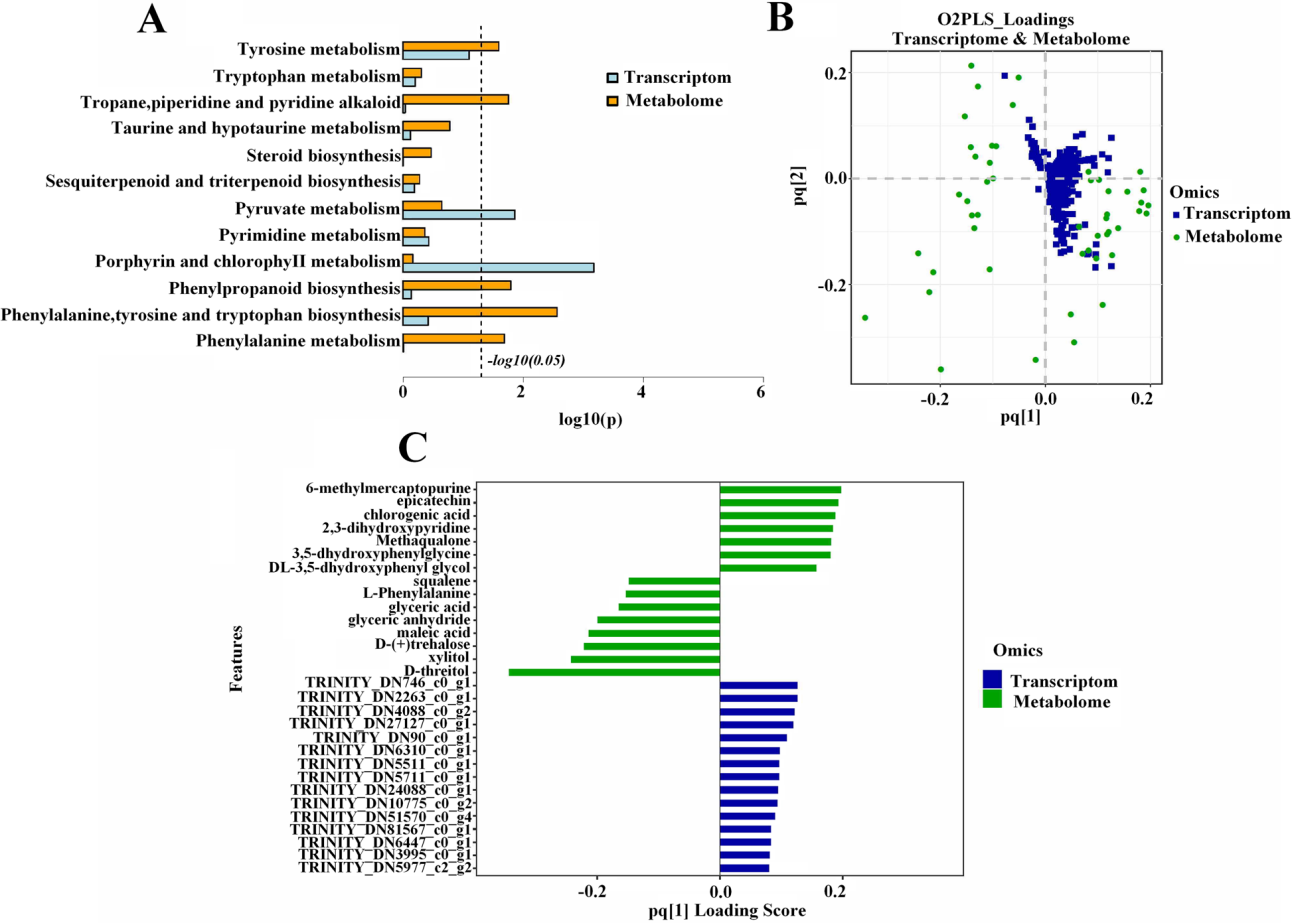
Correlation analysis between metabolites and transcripts. **A** Histogram of the KEGG pathway enriched by the differentially expressed genes and metabolites between CLH and CLL. The ordinate represents the pathway name, and the abscissa represents the pathway to impact. **B** Assess the intrinsic correlation between the metabolites and transcripts by the two-way orthogonal partial least squares (O2PLS) method. Solid blue squares represent genes and solid green dots represent metabolites. The abscissa and ordinate represent the combined loading value, each gene/metabolite has a relative coordinate point in pq1 and pq2, p represents the loading value of the gene, and q represents the loading value of the metabolite. **C** The top15 differential expresses gene and differential metabolite loading histogram. Blue bars represent genes and green bars represent metabolites. The abscissa represents the combined loading value pq1, and the ordinate represents the differential metabolite/differential gene

Correlation analysis between metabolites and transcripts of different varieties was performed to gain insight into the regulatory network of secondary metabolism of *C*. *longepaniculatum*. We conducted a joint KEGG pathway enrichment analysis, and identical pathways were enriched in the transcriptome and metabolome. A histogram was construct to visualize the enrichment degree of pathways with both DEGs and DAMs (Fig. 4A). The results showed that three KEGG pathways were related to phenylalanine metabolism: phenylalanine metabolism (map00360); phenylalanine, tyrosine and tryptophan biosynthesis (map00400); and phenylpropanoid biosynthesis (map00940). According to the results, 7 DEGs involved in the amino acid pathway (3) and the secondary metabolism (4) pathway were related to phenylalanine metabolism. After analysis of the secondary metabolites, 9 metabolites were found to be associated with phenylpropanoid biosynthesis.

We used two-way orthogonal partial least squares (O2PLS) to evaluate the intrinsic correlation between the transcriptomic and metabolomic data, calculated the score of each sample and obtained a joint score. Then, the load value of each gene and metabolite was calculated to obtain a load map (Fig. 4B). A joint score plot was constructed to visualize the relationship between the two data matrices, and metabolites/genes with high loading values were considered necessary for the similarity of the two data sets. Finally, the absolute values of the load value of the top 15 DAMs/DEGs were selected to construct a histogram (Fig. 4C). The correlation analysis showed that transcripts and metabolites were related to phenylalanine metabolic pathways.

### Confirmation of DEGs via qRT-PCR

**Fig. 5:**
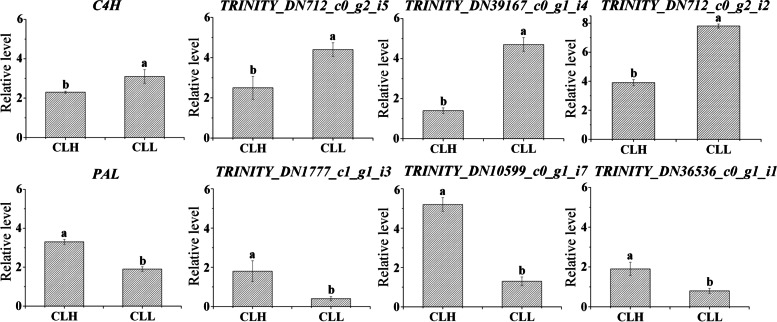
qRT-PCR validation of the relative expression levels of 8 selected genes from different varieties of *C*. *longepaniculatum*

To validate the reliability and stability of the RNA sequencing (RNA-seq) data of the DEGs, 8 DEGs were selected for qRT-PCR validation. Among them, four were upregulated in CLH (Fig. 5). The results suggested that the transcriptomic data were reliable.

## Discussion

**Fig. 6:**
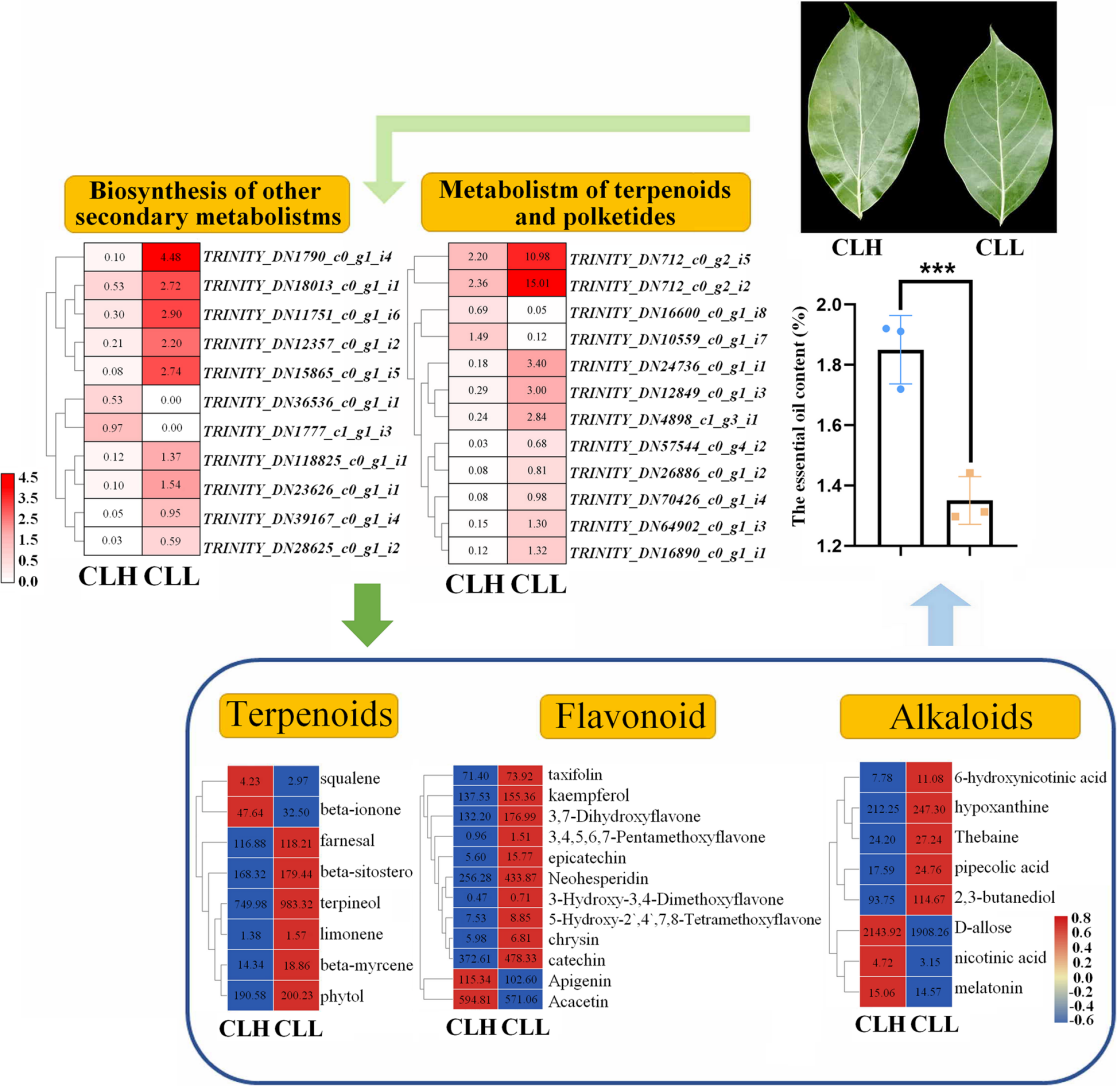
The accumulation of secondary metabolites in different varieties of *C. longepaniculatum*

Between the two varieties, 49 (26 downregulated and 23 upregulated) DAMs, 12 flavonoids, 8 terpenoids and 8 alkaloids were identified. Combined transcriptome and metabolome analyses revealed a strong correlation between metabolite content and gene expression (Fig. 6). We identified 12 flavonoids in *C*. *longepaniculatum* leaves (Table 4). Flavonoids are low-molecular-weight secondary metabolites produced by plants [28]; are present in all plant parts; and show a variety of functions, including antioxidant activity [29, 30], UV protection [31], pathogen protection and signalling [32, 33]. Constituting a large group of plant secondary metabolites, flavonoids are derived from the phenylpropanoid pathway [34]. Flavonoids, which include, flavones, flavonols, flavan-3-ols, flavanones, isoflavanones, isoflavans, and pterocarpans, have diverse structures. Previous studies suggest that apigenin [35], chrysin [36], neohesperidin [37], epicatechin [38], catechin [39], and kaempferol [40, 41] are bioactive substances that have antioxidant, antibacterial, antifungal, and antitumor activities and anti-inflammatory properties. Therefore, we believe that the flavonoids in *C*. *longepaniculatum* leaves are related to the plant stress response. It may be that endophytic fungi [27] cause the accumulation of flavonoids in the leaves of *C*. *longepaniculatum*, but we believe that the accumulation of flavonoids is caused by light. There is very important evidence in that 82 DEGs were mapped to 3 KEGG pathways that were related to light. We also found that the KEGG oxidative phosphorylation pathway was enriched in DAMs. Therefore, we speculate that different varieties of *C. longepaniculatum* have different responses to light, which results in differences in flavonoid contents. Nonetheless, this hypothesis needs further experimental confirmation.

PMFs constitute a unique class of flavonoids that are considered the biologically active constituents in citrus fruits [42]. We identified 4 kinds of PMFs from the leaves of *C. longepaniculatum*; this is the first report on the isolation of PMFs from *C. longepaniculatum*. PMFs can be used for anti-inflammatory, antiatherogenic, antiangiogenic and anticancer activities [43, 44], and different PMF structures lead to differences in biological activity [45]. By comparison, the PMFs identified in the present study are different from those in citrus, and the biological activities of PMFs from *C. longepaniculatum* need to be studied. Some studies have pointed out that citrus PMFs can affect the modulation of gut microbiota [10], and others have pointed out that microbial community composition and diversity can affect secondary metabolite content in leaves of *Cinnamomum camphora* [46]. We speculate that there is a complex relationship between PMFs and endophytic fungal communities of *C. longepaniculatum*.

A total of 7 classes and 8 kinds of terpenoids were identified in our research (Table 4). These components are largely consistent with the findings of previous studies [13, 46, 47]. Owing to their pleasant fragrance, unique biological activities and favourable physicochemical properties, terpineol [48, 49], eucalyptol [50], limonene [51], and squalene [52] have strong antioxidant properties and are widely applied in foods, pharmaceuticals, cosmetics, biomaterials, and biofuels [53, 54]. Among the terpenoids we identified, the contents in CLL were higher than those in CLH, except for squalene and beta-ionone; squalene synthesis occurs downstream of terpenoid synthesis. This difference between varieties may be because more substances may be converted to other terpenes, such as terpineol, in CLL and because less squalene is formed downstream. We analysed 196 terpenoids from fresh leaves of *C. longepaniculatum* via GC–MS. However, other researchers [19] used water extracts to identify 23 compounds that were detected from *C. camphora* leaves. Therefore, different extraction methods could affect the identification of substances.

In recent years, much research has involved the use of transcriptomic data to investigate the key regulatory genes related to terpenoid biosynthesis in *C. camphora* [21, 35]. Many terpenoid synthases have been isolated and characterized from various plant species [12]. In our research, our transcriptome analysis revealed that 23 DEGs were related to secondary metabolites, of which 12 DEGs were related to the metabolism of terpenoids and polyketides. The genes encoding S-(+)-linalool synthase (*TRINITY_DN10559_c0_g1*) and geranylgeranyl diphosphate synthase (*TRINITY_DN70426_c0_g1*) were differentially expressed in the different varieties (Fig. 6). The DEGs were all related to these two pathways, indicating that the essential oils in *C. longepaniculatum* metabolize are produced through both the MVA and the MEP pathways, which is consistent with the results of Chandran’s study [55]. Interestingly, a large number of DEGs were related to photosynthesis and oxidative phosphorylation. Like for flavonoids, we speculate that differences in light responses between the different varieties led to differences in terpenoid metabolism. *C. longepaniculatum* leaves synthesize a large number of secondary substances, such as flavonoids and terpenoids, to cope with light stress.

## Conclusions

In summary, the different varieties of *C. longepaniculatum* differ in essential oil content and phenotype. By performing transcriptome and metabolome analyses, we investigated the accumulation of secondary metabolites in different varieties of *C*. *longepaniculatum*. Of the 34,503 total genes discovered, we identified 1486 DEGs. KEGG analysis showed that phenylpropanoid, tryptophan biosynthesis and terpenoid pathways were significantly altered between the two varieties. In total, we detected 196 metabolites from *C*. *longepaniculatum*. Between the two varieties, 49 (26 downregulated and 23 upregulated) DAMs, and we identified 12 flavonoids, 8 terpenoids and 8 alkaloids. The finding of a high correlation between metabolite content and gene expression combined transcriptome and metabolome analyses. We speculate that light may cause differences in secondary metabolites and phenotypes in the leaves of *C. longepaniculatum*.

## Methods

### Plant materials

The two varieties of *C. longepaniculatum* (Gamble) N. Chao ex H. W. Li were identified by Professor Ruizhang Feng (Yibin University, Yibin, PR China). Voucher specimens (CLH, 20171026YBU016; CLL, 20171026YBU014) were deposited in the herbarium of the Faculty of Agriculture, Forestry and Food Engineering, Yibin University, Yibin, PR China. The leaves of different *C. longepaniculatum* varieties (CLH, CLL) were used as experimental materials. The two varieties were propagated from one mother tree via cuttings. *C. longepaniculatum* was planted on Hongyan Mountain (27°50′ N, 105° 20′ E) in Yibin, Sichuan Province, PR China. No specific permits were required to collect the plant materials because Hongyan Mountain is a germplasm resource nursery of Yibin University. The leaves used for the experiments were collected in November 2019, November 2020 and November 2021. For each variety, 10 healthy plants that have been growing for 20 years were randomly selected. One hundred functional leaves were selected from the sun-facing and shaded sides of the trees. All the leaves of the same variety were mixed and then divided into three groups as 3 biological replicates. Some of the fresh materials were cut into small pieces, immediately frozen in liquid nitrogen and stored at − 80 °C until further transcriptome and metabolome analysis. The others materials were air dried at room temperature and then subjected to distillation to obtain essential oils.

### Essential oil isolation

The leaves used for essential oil extraction were collected in November 2019, November 2020 and November 2021. After air drying, 100 g of leaves was put into a steam distillation instrument for 1.5 h to isolate essential oils. The isolation of essential oils from each sample was repeated three times. The essential oil yield was estimated on a dry weight basis (w/w).

### Cytological observations of leaves

Leaves of different varieties were harvested in November 2020, kept in formaldehyde-acetic acid-ethanol (FAA) (70% ethanol:acetic acid:38% methanol = 90:5:5), and then embedded in paraffin after dehydration through a series of ethanol concentrations according to the manufacturer’s protocol. Cross-sections of 2 to 5 mm thickness were cut with a microtome (Leica RM2235). The cross-sections were double dyed with safranine and fast green and covered with a slide cover. The sections of the leaves were observed under a microscope (Motic BA200). The leaf cross-sectional area and leaf thickness were subsequently determined with ImageJ (image processing and analysis in Java).

### RNA extraction, library construction and sequencing of fresh leaves

The leaves of different varieties collected in November 2020 were used for RNA-seq. Total RNA was obtained using an RNA purification reagent for plant tissue (Invitrogen, Carlsbad, CA, USA). Three biological replicates were sampled per variety. Six RNA-seq transcriptome libraries were prepared using an Illumina TruSeq™ RNA Sample Preparation Kit (San Diego, CA). Poly(A) mRNA was purified from the total RNA using oligo-dT-attached magnetic beads and then fragmented with fragmentation buffer. Taking these short fragments as templates, double-stranded cDNA was synthesized using a SuperScript Double-Stranded cDNA Synthesis Kit (Invitrogen, CA) with random hexamer primers (Illumina). Then, the synthesized cDNA was subjected to end repair, phosphorylation and polyadenylation according to Illumina’s library construction protocol. Libraries were size selected for cDNA target fragments of 200–300 bp on 2% low-range ultra-agarose followed by PCR amplification using Phusion DNA polymerase (New England Biolabs, Boston, MA), with 15 PCR cycles. After quantification by a TBS-380 instrument, 6 RNA-seq libraries were sequenced in a single lane on an Illumina HiSeq Xten/NovaSeq 6000 sequencer (Illumina, San Diego, CA) for 2 × 150 bp paired-end reads.

### De novo assembly and annotation

The raw paired-end reads were trimmed and subjected to quality control by SeqPrep (https://github.com/jstjohn/SeqPrep) and Sickle (https://github.com/najoshi/sickle), with the default parameters. Then, clean data from the samples (*C*. *longepaniculatum*) were utilized to perform de novo assembly with Trinity (http://trinityrnaseq.sourceforge.net/) [56]. All the assembled transcripts were searched via BLASTX against the sequence information within the NCBI protein nonredundant (NR), COG, and KEGG databases to identify the proteins whose sequences were most similar to those of the given transcripts to retrieve their functional annotations, and a typical cut-off E-value of less than 1.0 × 10^− 5^ was set. The BLAST2GO (http://www.blast2go.com/b2ghome) [57] program was used to obtain GO annotations of unique assembled transcripts for describing biological processes, molecular functions and cellular components. Metabolic pathway analysis was performed using the KEGG database (http://www.genome.jp/kegg/) [58].

### Differential expression analysis and functional enrichment

To identify DEGs between the two varieties, the expression level of each transcript was calculated according to the transcripts per million reads (TPM) method. Afterwards, RSEM (http://deweylab.biostat.wisc.edu/rsem/) [59] was used to quantify gene abundance. Specifically, differential expression analysis was performed using DESeq2 [60]/EdgeR [61] with a Q value ≤0.05. Genes with |log2(FC)| > 1 and Q value <= 0.05 (DESeq2 or EdgeR)/Q value <= 0.001 (DEGseq) were considered to be significantly differentially expressed. In addition, functional enrichment analysis via the GO and KEGG databases was performed to identify which DEGs were significantly enriched in GO terms and metabolic pathways with a Bonferroni-corrected *P* value of ≤0.05 compared with the whole-transcriptome background. GO functional enrichment and KEGG pathway analysis were carried out by GOATOOLS (https://github.com/tanghaibao/Goatools) and KOBAS (http://kobas.cbi.pku.edu.cn/home.do), respectively [62, 63].

### Extraction of metabolites from fresh leaves and GC–MS analysis

The leaves of different varieties collected in November 2020 were used for metabolite extractions. Three biological replicates were sampled per variety. Fifty milligrams of leaf tissue was extracted in 500 μL of 75% methanol, 2% L-2 chlorophenylalanine and 200 μL of chloroform were added, and the mixture was homogenized at 50 Hz for 3 min at − 10 °C. The mixture was then subjected to ultrasonic treatment at 40 kHz for 10 min at 5 °C after vortexing for 30 s, the process of which was repeated 3 times. After being allowed to settle at − 20 °C for 30 min, the sample was centrifuged at 12000 rcf at 4 °C for 20 min. The supernatant was then vacuum dried. A total of 80 μL of methoxyamine hydrochloride (15 mg/mL in pyridine) was added to the sample, which was then shaken for 2 min and incubated at 37 °C for 90 min. Finally, 80 μL of bis-(trimethylsilyl) trifluoroacetamide (BSTFA) with 1% trimethylchlorosilane (TMCS) and 20 μL of n-hexane were added, after which the sample was stored at 70 °C for 60 min after shaking for 2 min. The samples were subsequently incubated at room temperature for 30 min and then analysed by GC–MS. An extract of each sample was prepared as a quality control (QC) sample. The QC samples were compiled and tested in the same manner as the analytic samples. To assess the repeatability of the analysis, the QC samples were injected at regular intervals (every 3 samples).

GC–MS analysis was conducted on an Agilent 8890B gas chromatograph coupled to an Agilent 5977B mass selective detector (inert electron impact ionization (EI) source and ionization voltage of 70 eV) (Agilent, USA). The gas chromatograph was equipped with an HP-5MS (30 m × 0.25 mm × 0.25 μm) capillary column, and the gas chromatograph column temperature was programmed to hold at 60 °C, increase to 310 °C at a rate of 8 °C/m and hold at the final temperature for 6 min. The gas chromatograph conditions included an inlet temperature of 310 °C, helium carrier gas at a constant flow rate of 1 mL/min, a resting oven temperature of 60 °C and a GC–MS transfer line temperature of 300 °C. After a sample injection of 1 μL, the sample was introduced in splitless mode with an inlet temperature of 260 °C. The ion source temperature was 230 °C, and the quadrupole temperature was 270 °C. Data acquisition was conducted in full-scan mode with a range of m/z 50–500.

### Metabolome: data processing and statistics

The GC–MS data were analysed with MassHunter Workstation Quantitative Analysis (version 10.0.707.0). An internal standard was used for data QC (reproducibility). Samples with metabolite features and a relative standard deviation (RSD) of QC > 30% were discarded. A multivariate statistical analysis was performed using ropls (version 1.6.2, http://bioconductor.org/packages/release/bioc/html/ropls.html). All of the metabolite variables were scaled to unit variances prior to conducting principal component analysis (PCA). VIPs were calculated via a OPLS-DA model, and *P* values were estimated with paired Student’s t test via single-dimensional statistical analysis. The metabolites that differentially accumulated between the two groups were mapped to their biochemical pathways through metabolic enrichment and pathway analysis based on a database search (KEGG; http://www.genome.jp/kegg/). Scipy.stats (a Python package) (https://docs.scipy.org/doc/scipy/) was exploited to identify a statistically significantly enriched pathway using Fisher’s exact test.

### Combined transcriptome and metabolome analyses

Pearson correlation coefficients were calculated for metabolome and transcriptomic data integration. Based on the Pearson correlation coefficient, correlations between genes and metabolites in samples can be measured, and the range of the correlation coefficient is (− 1, + 1). A correlation coefficient less than 0 indicates a negative correlation; when it is greater than 0, it indicates a positive correlation; and when it is equal to 0, it indicates no correlation. The threshold of the correlation coefficient in this study was ±0.8, and the correlation was significant (*P* < 0.05). The DEGs and DAMs related to essential oil biosynthesis were used to construct coexpression networks, and the candidate target genes were visualized by Cytoscape (version 3.7.2, USA).

### Validation of RNA-Seq data by qRT-PCR

Total RNA was isolated from approximately 100 mg of fresh leaves of the different varieties of *C*. *longepaniculatum*, which were collected in November 2020, using an RNAprep Pure Plant Plus Kit (TIANGEN, Beijing, China). One microgram of total RNA was subjected to reverse transcription using a SYBR Green PCR Master Mix (TaKaRa) Kit with gDNA Eraser (Perfect for Real-Time). Real-time PCR was carried out by using SYBR Premix Ex Taq II (TaKaRa) on an ABI StepOne™ Plus Real-Time PCR System (Roche, Switzerland). All the primers used for qRT–PCR are listed in Table S5. Relative gene expression was calculated using the 2^-ΔΔCt^ method, and owing to its stable expression via PCR amplification, the *ACT* (KM086738.1) gene was used as a reference control gene [22].

### Statistical analysis

The control and treatment groups were analysed for statistically significant differences using one-way ANOVA followed by Duncan’s multiple comparisons test. All the calculations were performed using SPSS software (version 21; IBM, Armonk, NY, USA), and all the results are presented as the mean ± SD of 3 independent biological replications. The treatment means were separated by Duncan’s multiple range test at a *P* value less than 0.01. We then used the min–max normalization method of TBtools [63] to analyse the transcriptomic and metabolomic data representing the expression values used in our heatmap.

## Supplementary Information

**Additional files 1: Fig. S1.** The Venn diagram analysis of genes expressed in different varieties.

**Additional files 2: Fig. S2.** The metabolites of different varieties analyzed by the KEGG pathways.

**Additional files 3: Table S1.** The fold change and *P*-value of DEGs.

**Additional files 4: Table S2.** The DEGs functional classification.

**Additional files 5: Table S3.** The significantly altered KEGG pathways.

**Additional files 6: Table S4.** The total metabolites from the leaf of C. *longepaniculatum*.

**Additional files 7: Table S5.** The primers used for qRT-PCR.

## Data Availability

The datasets generated and analyzed during the current study are available in the Biological Research Project Data (BioProject), National Center for Biotechnology Information (NCBI) repository, accession: PRJNA804339.

## Abbreviations

DEGs: Differential expression genes
DAMs: Differentially accumulated metabolites
GO: Gene Ontology
KEGG: Kyoto encyclopedia of genes and genomes
qRT-PCR: Quantitative real-time PCR
PMFs: Polymethoxyflavones
