# Supplementary figures and images for: Transcriptome and metabolome reveal the accumulation of secondary metabolites in different varieties of Cinnamomum longepaniculatum
Source: BMC Plant Biol. 2022 May 18;22:243. doi: 10.1186/s12870-022-03637-2 (PMC9116011; doi:10.1186/s12870-022-03637-2)

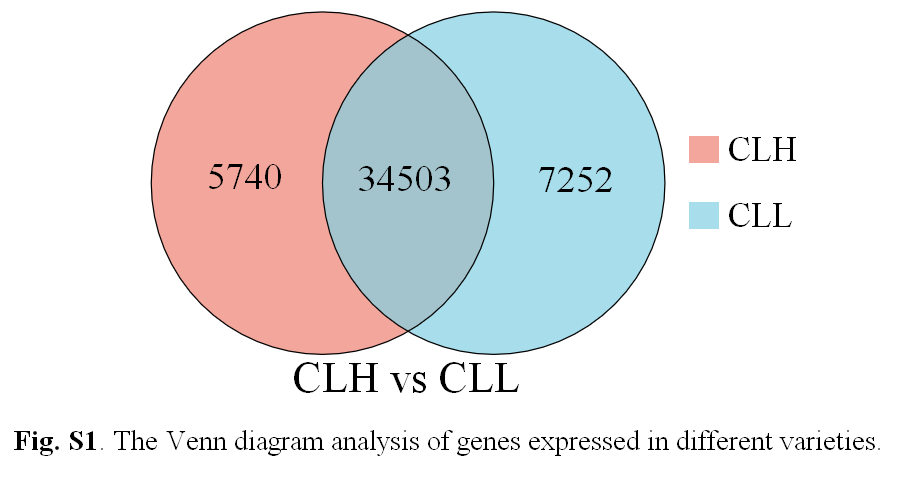

Supplement: Supplementary file 1 — Additional files 1: Fig. S1. The Venn diagram analysis of genes expressed in different varieties. [file 12870_2022_3637_MOESM1_ESM.tif]

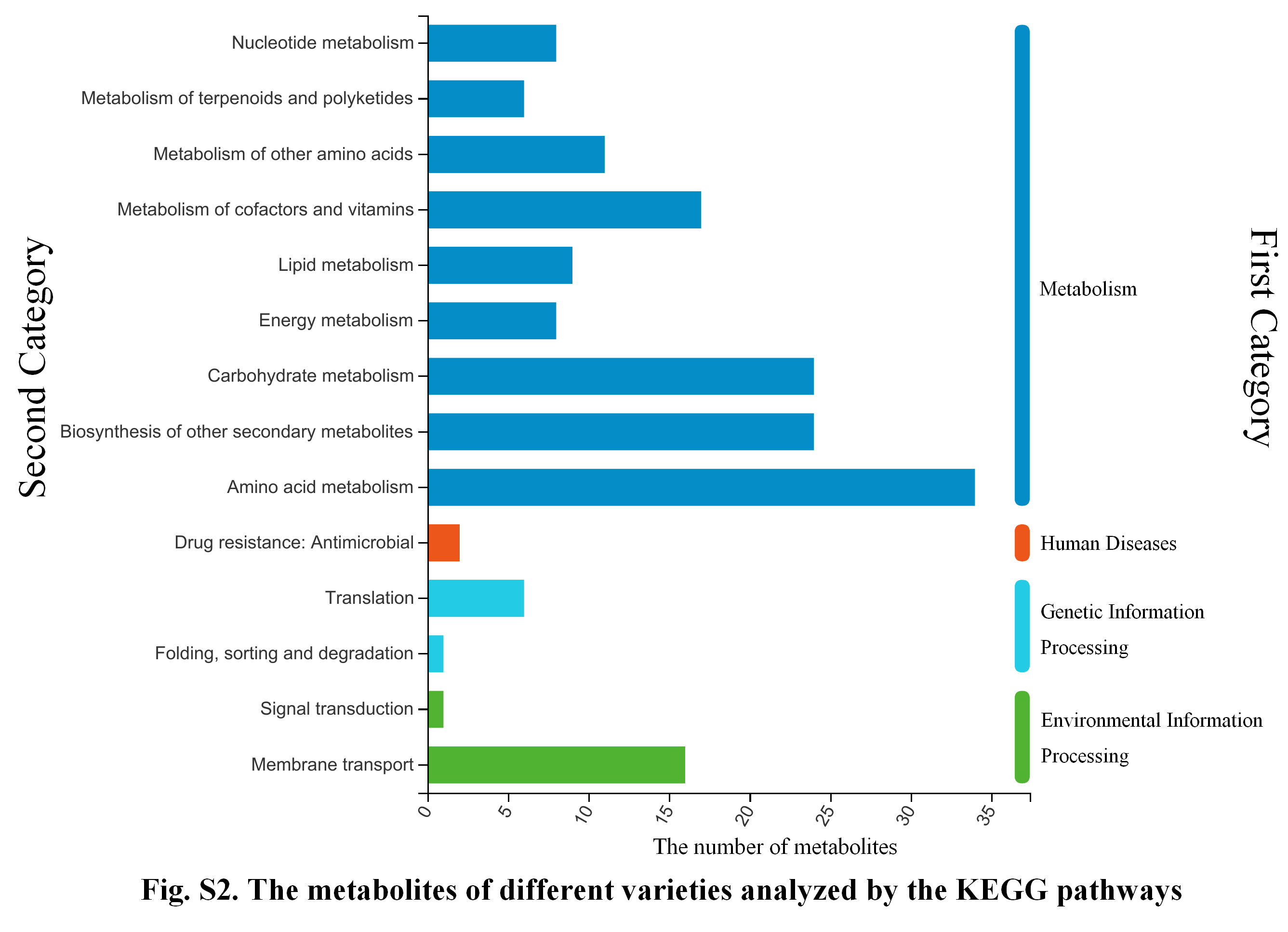

Supplement: Supplementary file 2 — Additional files 2: Fig. S2. The metabolites of different varieties analyzed by the KEGG pathways. [file 12870_2022_3637_MOESM2_ESM.tif]
